# Successful treatment of eosinophilia associated with dialysis‐related renal cancer with radical nephrectomy

**DOI:** 10.1002/iju5.12729

**Published:** 2024-04-24

**Authors:** Yuta Goto, Daichi Tamura, Tomohiko Matsuura, Ei Shiomi, Daiki Ikarashi, Shigekatsu Maekawa, Renpei Kato, Mitsugu Kanehira, Wataru Obara

**Affiliations:** ^1^ Department of Urology Iwate Medical University Shiwa‐gun Iwate Japan

**Keywords:** dialysis, eosinophilia, nephrectomy, renal cancer, solid tumor

## Abstract

**Introduction:**

Secondary eosinophilia due to solid tumors is a rare case. This is the first study to report secondary eosinophilia due to renal cancer in a patient on dialysis.

**Case presentation:**

A 70‐year‐old man, on long‐term hemodialysis was incidentally detected with right renal cancer, and workup performed revealed eosinophilia. Allergic symptoms caused by hemodialysis were initially considered; however, treatment did not lead to any improvement in eosinophilia. Therefore, nephrectomy for renal cancer was performed. The resolution of symptoms and eosinophilia after surgery suggested renal cancer as the cause of eosinophilia.

**Conclusion:**

As demonstrated in this patient with dialysis‐related renal cancer, eosinophilia associated with solid tumors may be addressed by treating the tumor.

**Abbreviations & Acronyms**
CTA membrane: cellulose triacetate membrane
GM‐CSF: granulocyte‐macrophage colony‐stimulating factor
IL‐5: interleukin 5
PMMA membrane: polymethylmethacrylate membrane
VEGF‐A: vascular endothelial growth factor A

**Keynote message**

Eosinophilia can occur secondary to solid tumors; however, secondary eosinophilia due to renal cancer in a patient on dialysis has not been previously reported. We experienced eosinophilia associated with dialysis‐related renal cancer and removal of the renal cancer by nephrectomy resolved eosinophilia.

## Introduction

Eosinophilia, defined as an eosinophil count of ≥500/μL, is often associated with allergic symptoms such as fever and cough.^1^, ^2^ In addition to primary eosinophilia, allergies, parasitic diseases, medications, autoimmunity, and solid tumors are common causes of secondary eosinophilia.^3^ Few studies have reported eosinophilia caused by solid tumors. Takatsu *et al*.^1^ found only 37 cases of solid tumor‐associated eosinophilia reported in Japan until 2021. Only four cases of eosinophilia secondary to renal cancer have been reported, including those in the English literature,^4^, ^5^ and secondary eosinophilia with dialysis‐related renal cancer has not been reported. Eosinophilia associated with solid tumors might be related to cytokine production from the tumor.^1^ Although some studies examined cytokine levels before treatment,^6^, ^7^ no study evaluated changes in cytokine levels after treatment in patients with solid tumor‐associated eosinophilia. We present a patient with eosinophilia associated with dialysis‐related renal cancer.

## Case report

A 70‐year‐old man experienced cardiac arrest during hemodialysis in another hospital was transferred to our hospital. He was on hemodialysis for 11 years due to end‐stage renal failure secondary to nephrosclerosis. He was also on prednisolone (2 mg/day) for 8 years due to eosinophilia of unknown cause, with eosinophil counts remaining within normal limits after treatment.

**Fig. 1:**
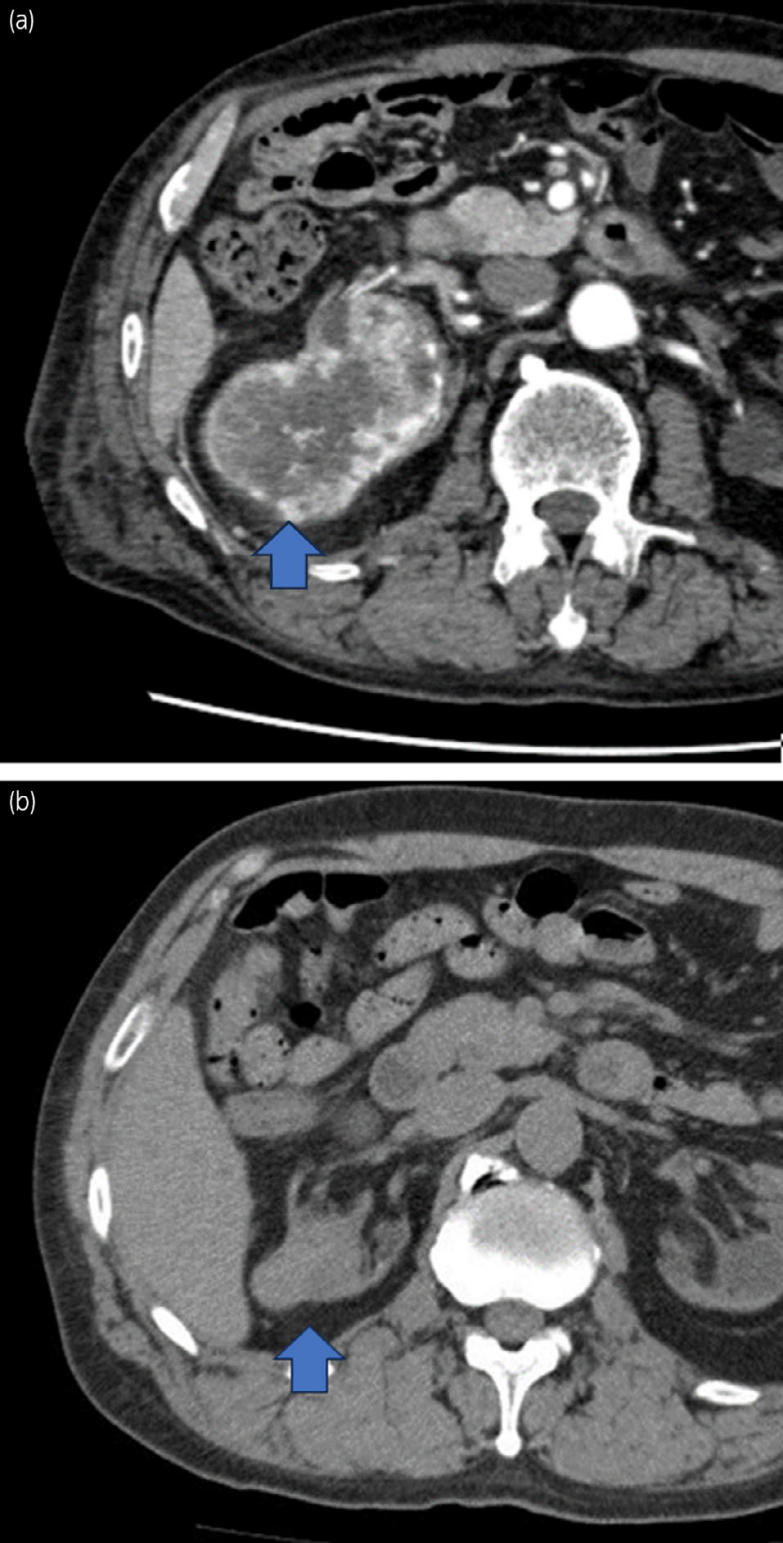
(a) Computed tomography image at admission showing a mass with fresh hematoma in the upper pole of the right kidney (blue arrow). (b) Computed tomography from 8 years earlier showing a 40‐mm internally heterogeneous mass in the upper pole of the right kidney (blue arrow).

**Fig. 2:**
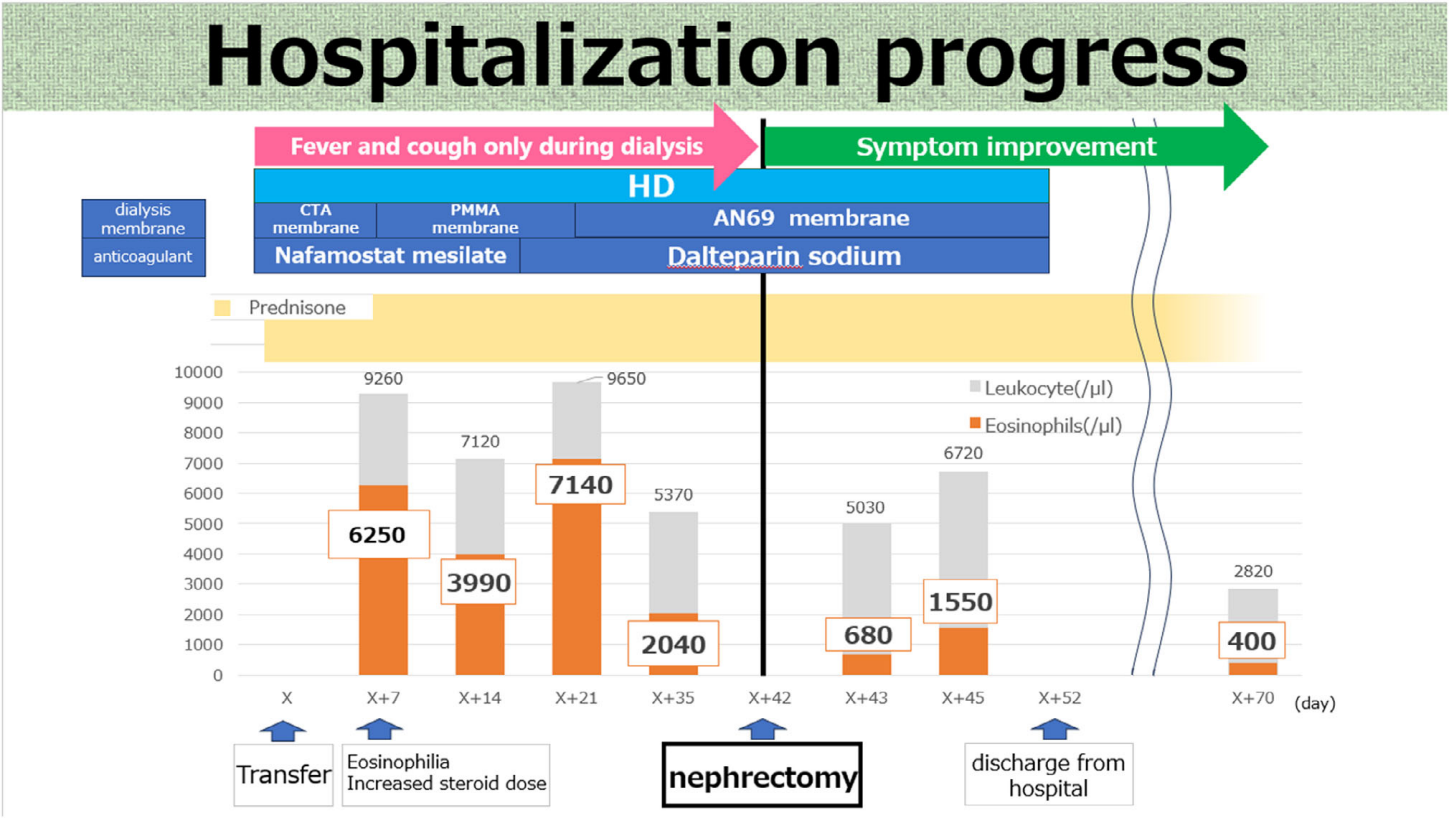
Changes in the eosinophil count, prednisolone, symptoms, dialysis membrane, anticoagulant of the patient during hospitalization.

Computed tomography at admission revealed a renal tumor with hemorrhage in the right kidney (Fig. 1a). Hemodialysis and tumor hemorrhage were considered to trigger hemodynamic breakdown and cardiac arrest. His condition improved with multidisciplinary treatment and he was transferred to our department for renal tumor treatment. Continuous hemodiafiltration was performed until his condition stabilized, and he was switched to intermittent hemodialysis after admission to our department. However, he developed fever and cough, and blood tests revealed eosinophilia (6250/μL). Dialysis‐related eosinophilia was considered. The dialysis membrane and anticoagulants were changed, and the steroid dose was increased, which did not improve the symptoms or eosinophilia. After excluding the potential causes of secondary eosinophilia other than hemodialysis, right renal cancer, was considered. He underwent right nephrectomy on the 42nd day after being transferred to our department. Pathologic evaluation led to the diagnosis of clear cell renal carcinoma, pT2a. Fever and cough during dialysis disappeared, and eosinophil count quickly decreased after surgery. He was discharged on the 52nd day after transfer to our department. He has been continuing hemodialysis without symptomatic flare‐up under the care of his previous physician (Fig. 2).

## Discussion

Our patient developed fever and cough during dialysis after the initiation of intermittent dialysis. Diagnostic workup identified eosinophilia, which had been managed until his current admission, and the relapse of eosinophilia was considered to cause his symptoms. Many studies report allergic reaction to the dialysate, dialysis membrane, or anticoagulants^8^, ^9^, ^10^ as a cause of eosinophilia. However, failure to resolve symptoms and eosinophilia after switching the dialysis membrane and anticoagulants led to the suspicion of renal cancer the cause. Indeed, the subjective symptoms and eosinophilia quickly disappeared after tumor removal. Retrospective evaluation of the computed tomography images from 8 years earlier revealed findings suggesting a mass lesion in the upper pole of the right kidney, the same location where the current tumor was observed (Fig. 1). These findings suggested that eosinophilia, which was considered to be due to an unknown cause at the time, might have been secondary to renal cancer.

**Fig. 3:**
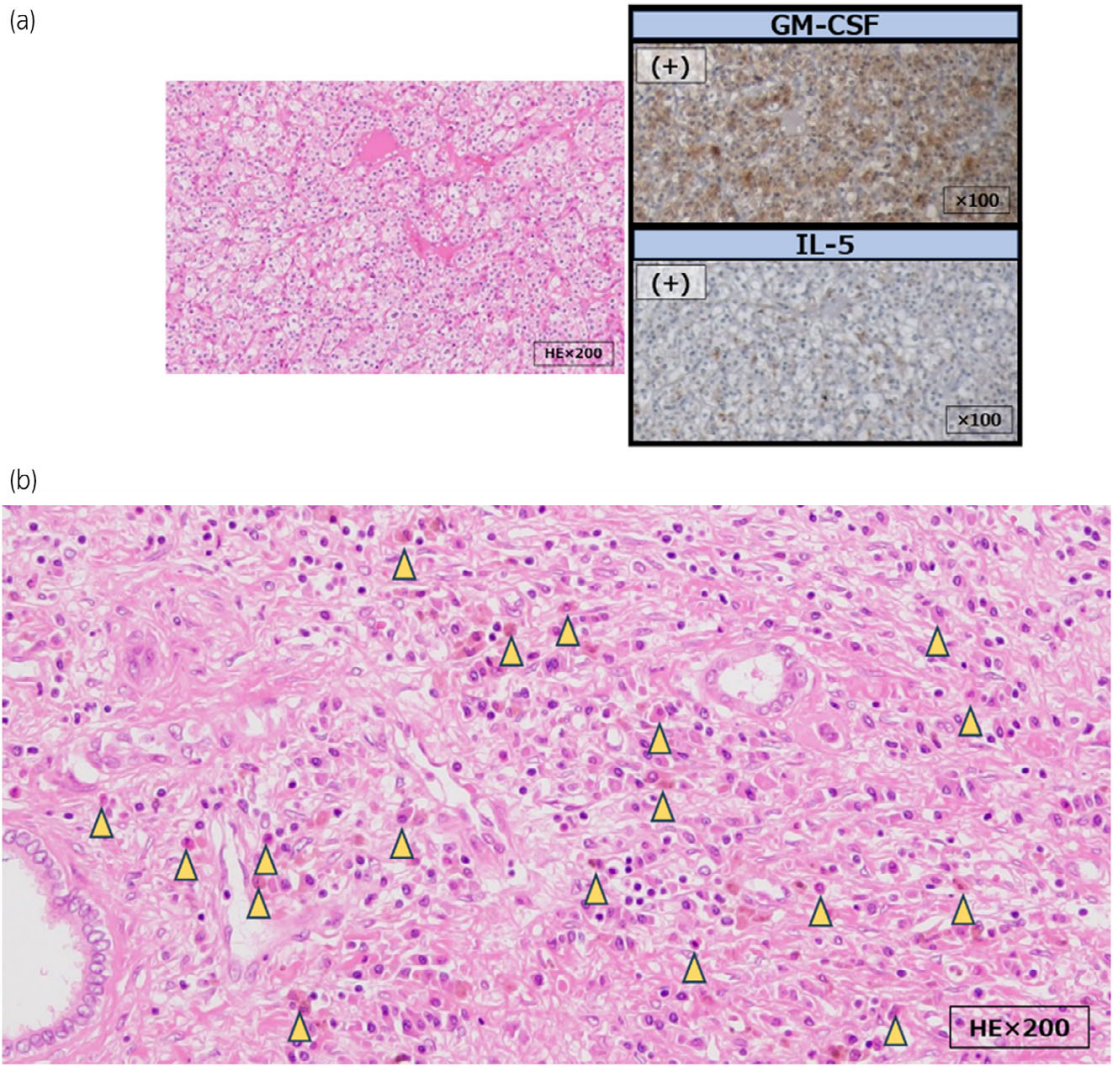
(a) Histopathologic evaluation of the tumor. Hematoxylin/eosin staining showing tumor cells with round nuclei and clear cytoplasm. Immunostaining showing positivity for GM‐CSF and IL‐5. (b) Histopathologic evaluation with hematoxylin/eosin staining showing eosinophilia in the renal parenchyma adjacent to the tumor (yellow triangles).

Eosinophil differentiation is induced by cytokines such as GM‐CSF and IL‐5. In particular, IL‐5 acts specifically on eosinophils and is involved in the final differentiation into eosinophils.^11^ Excessive production of these cytokines by tumor cells is considered to lead to eosinophilia.^1^ Furthermore, Gerharz *et al*.^12^ reported that GM‐CSF is usually positive in renal cancer but no expression of IL‐5 was observed. In our case, GM‐CSF and IL‐5 in the tumor tissue were positive, and an increase in eosinophils was observed in the renal parenchyma (Fig. 3). Therefore, we considered that the presence of IL‐5 is important in eosinophilia associated with renal cancer.

We suspected renal cancer as the cause of eosinophilia and expected that preoperative serum cytokine levels and the eosinophil count would be high and that both would quickly decrease after surgery. However, serum levels of several cytokines, including GM‐CSF and IL‐5, were within the normal range before surgery and increased on postoperative Day 1 (Table 1). Furthermore, the eosinophil count decreased to the normal range on postoperative Day 1, followed by a slight increase thereafter. We suspected eosinophilia associated with dialysis in the present case. Accordingly, we changed the dialysis membrane and finally used AN69 membrane with cytokine adsorption ability. This usage may have prevented the increase in cytokine levels before surgery. Additionally, we considered that performing continuous hemodiafiltration using the AN69ST membrane with cytokine adsorption ability at the beginning of his transfer to our hospital removed the cytokines continuously and the symptoms did not develop. Regarding the changes in the eosinophil count and cytokine levels after surgery, the surgical removal of eosinophils from the renal tissue might have led to increased GM‐CSF and IL‐5 production from T and mast cells in an attempt to maintain eosinophil homeostasis. Additionally, as eosinophils differentiate from progenitor cells to mature eosinophils over several days,^11^ we concluded that the increase in eosinophils occurred because of the increase in cytokines after surgery. Ultimately, because the negative feedback suppressed cytokine production, it was thought that eosinophil count stabilized to within the normal range.

**Table 1:** Changes in cytokine levels after surgery

	Preoperative (pg/mL)	Postoperative (pg/mL)
GM‐CSF	8.26	**27.06**
IL‐3	Below the limit of detection	Below the limit of detection
IL‐5	12.8	**441.37**
IL‐8	16.03	16.15
IL‐13	71.95	64.61
VEGF‐A	182.5	166.1

Immunostaining for GMCSF and IL5 are shown in bold to emphasize that they were elevated postoperatively.

In patients with solid tumor‐associated eosinophilia, treatment of the tumor, including surgery and chemotherapy, has been demonstrated to resolve eosinophilia.^1^ In this case, the patient was able to discontinue prednisolone postoperatively, proving that treatment of the primary tumor can improve eosinophilia associated with dialysis‐related renal cancer.

In this case, improvement of symptoms and normalization of the eosinophil count were observed following renal cancer treatment, although no association between cytokine levels and cancer treatment was observed. Although no specific time period until eosinophil decline has been reported, most studies with eosinophilia associated with solid tumors reported that eosinophil counts decreased 1 month after treatment, and future studies evaluating the relationship between cytokines and eosinophil count should consider the timing of measurements.

## Conclusion

In patients on hemodialysis, eosinophilia is primarily considered to be due to dialysis‐related factors, which may delay the workup for solid tumors. Solid tumors should be considered potential contributing factors to eosinophilia.

## Author contributions

Yuta Goto: Conceptualization; data curation; formal analysis; investigation; methodology; project administration; writing – original draft. Daichi Tamura: Methodology; project administration. Tomohiko Matsuura: Supervision; writing – review and editing. Ei Shiomi: Investigation; methodology; project administration. Daiki Ikarashi: Investigation; methodology; project administration. Shigekatsu Maekawa: Investigation; methodology; project administration. Renpei Kato: Investigation; methodology; project administration; supervision. Mitsugu Kanehira: Investigation; methodology; project administration; supervision. Wataru Obara: Supervision; writing – review and editing.

## Conflict of interest

The authors declare no conflict of interest.

## Approval of the research protocol by an Institutional Reviewer Board

N/A.

## Informed consent

Written informed consent for publication was obtained from the patient.

## Registry and the Registration No. of the study/trial

Not applicable.

## References

[1] Takatsu Y , Moriguchi Y . A case of advanced colon cancer with two episodes of hyper‐eosinophilia. Jpn. Soc. Coloproctol. 2021; 74: 168–174.

[2] Waku T , Sonobe H . A case of anaplastic thyroid cancer presented with eosinophilia. Off. J. Jpn. Assoc. Endoc. Surg. Jpn. Soc. Thyroid Surg. 2017; 34: 204–208.

[3] Chihara J , Ito W . Classification and diagnosis of diseases with hypereosinophilia. Jpn. Soc. Allergol. 2011; 60: 676–681. 21709434

[4] Wei Y‐B , Yan B , Yin Z *et al*. Chromophobe renal cell carcinoma associated with eosinophilia: a report of three cases. Exp. Ther. Med. 2014; 8: 91–94. 24944603 10.3892/etm.2014.1725 PMC4061223

[5] Tilman T , Stefan W , von Weyhern CH *et al*. Severe paraneoplastic hypereosinophilia in metastatic renal cell carcinoma. BMC Urol. 2012; 12: 1–7. 22436420 10.1186/1471-2490-12-7 PMC3348004

[6] Takeyama S , Kimura M , Miura T , Fukumori Y , Yasui H , Ueda T . A case of pancreatic acinar cell carcinoma producing granulocyte‐macrophage colony‐stimulating factor (GM‐CSF) with eosinophilia. Jpn. Soc. Intern. Med. 2018; 107: 276–283.

[7] Higashiyama A , Kudo M , Nagasako T *et al*. Successful chemotherapy of carcinomatosis of the bone marrow with disseminated intravascular coagulation from a rectal carcinoma found by eosinophilia. Jpn. Soc. Gastroenterol. 2011; 108: 1244–1251. 21737977

[8] Koinuma K , Kimura H , Tsukida M , Mishima K . A case in which an allergy was suspected to have been caused by a bicarbonate dialysate containing acetate. Jpn Soc. Dial. Ther. 2018; 51: 545–550.

[9] Adachi T , Machida S , Sasaki S *et al*. A case of strongyloidiasis detected while searching for the cause of eosinophilia in a patient undergoing hemodialysis. Jpn Soc. Dial. Ther. 2015; 48: 657–662.

[10] Saito O , Kusano E , Akai Y *et al*. Nafamostat mesilate induced eosinophilia; mechanism of eosinophilia and its relationship to IL‐2 and IL‐3. Jpn Soc. Dial. Ther. 1997; 30: 205–210.

[11] Ackerman SJ , Bochner BS . Mechanisms of eosinophilia in the pathogenesis of hypereosinophilic disorders. Immunol. Allergy Clin. N. Am. 2007; 27: 357–375. 10.1016/j.iac.2007.07.004 PMC2064859 17868854

[12] Gerharz CD , Reinecke P , Schneider EM , Schmitz M , Gabbert HE . Secretion of GM‐CSF and M‐CSF by human renal cell carcinomas of different histologic types. Urology 2001; 58: 821–827. 11711375 10.1016/s0090-4295(01)01371-1

